# Salicylic Acid Priming Regulates Stomatal Conductance, Trichome Density and Improves Cadmium Stress Tolerance in *Mentha arvensis* L.

**DOI:** 10.3389/fpls.2022.895427

**Published:** 2022-07-05

**Authors:** Abbu Zaid, Firoz Mohammad, Kadambot H. M. Siddique

**Affiliations:** ^1^Plant Physiology and Biochemistry Section, Department of Botany, Aligarh Muslim University, Aligarh, India; ^2^Department of Botany, Government Degree College Doda, Doda, India; ^3^The UWA Institute of Agriculture, The University of Western Australia, Perth, WA, Australia

**Keywords:** cadmium stress, *Mentha arvensis*, photosynthesis, salicylic acid, secondary metabolites

## Abstract

The application of phytohormones through seed priming could enhance quality of important medicinal and aromatic plants (MAPs) under heavy metal stress. We evaluated the potential of salicylic acid (SA) priming for overcoming the adverse effects of cadmium stress in *Mentha arvensis* L. plants. Suckers of plants were primed with SA before transplanting them into soil. At 30 days after transplanting, two doses (50 and 100 μm) of CdCl_2_ were applied to the soil. Both Cd treatments altered plant growth, photosynthetic pigments, leaf gas exchange attributes, and mineral nutrient contents. The 50 and 100 μm Cd treatments increased endogenous Cd content by 97.95 and 98.03%, electrolyte leakage (EL) by 34.21 and 44.38%, hydrogen peroxide (H_2_O_2_) by 34.71 and 55.80%, malondialdehyde (MDA) by 53.08 and 63.15%, and superoxide content (O_2_^–•^) by 24.07 and 38.43%, respectively. Cd triggered the up-regulation of antioxidant enzyme activities (superoxide dismutase, SOD; catalase, CAT; ascorbate peroxidase, APX; and glutathione reductase GR) and increased osmolyte biosynthesis and, interestingly, secondary metabolite (SM) accumulation. The presence of SA and Cd had an additive effect on these parameters. Nevertheless, plants primed with SA regulated stomatal conductance under Cd stress. SA priming to menthol mint plants under Cd stress overcome the effects of Cd stress while increasing SMs.

## Introduction

Heavy metal (HM) contamination is ubiquitous in all the spheres of the environment, *viz.* biosphere, lithosphere, atmosphere, hydrosphere, and anthroposphere. The plant–soil interface acts as a channel for different HMs to circulate into different environmental compartments and ultimately reach humans and plants through the food chain. In particular, cadmium (Cd) is a potent and noteworthy environmental contaminant. In most soils, Cd is present as a trace element. However, due to high industrialization and sophisticated anthropogenic activities, such as the discharge of sewage sludge, mining, phosphorous fertilizers, and industrial effluents, excessive amounts of Cd are bio-available in vast tracts of lands (Zhang et al., 2009; He et al., 2015; Gao et al., 2018; Zaid et al., 2019a; Shah et al., 2020a,b). Cd pollution is the representative HM contamination in soil, as Cd^2+^ ions are readily absorbed by crop plants (DalCorso et al., 2008; Rizwan et al., 2018). In plants, Cd can close stomata, generate reactive oxygen species (ROS), oxidize membranes, lipids and proteins, and decrease photosynthetic potential, subsequently disturbing plant growth and development (Zaid and Mohammad, 2018; Bari et al., 2019; Pourghasemian et al., 2019).

Menthol mint (*Mentha avensis* L.) is a wild mint variety widely cultivated for its phytopharmaceutical importance (Nigam et al., 2019). Soil Cd enrichment is posing a major threat to its productivity by inducing oxidative stress. *M. arvensis* L. owes its aromatic properties to the presence of an essential oil (EO) rich in SMs and volatile constituents, including menthol, menthone, methyl acetate, and carvacrol, that impart its characteristic mint flavor (Tiwari, 2016). Advances in *M. arvensis* trichome research, stomatal conductance, and SMs biosynthesis under Cd stress have received little attention. Till date, no study has reported plant growth regulator (PGR)-mediated mechanisms of action for enhancing tolerance to Cd stress and SM production in *M. arvensis*.

Plant stress physiologists have attempted to reduce Cd contamination in soils and crop plants using various organic and inorganic molecules (Rizwan et al., 2016, 2017, 2019b; Zaid and Mohammad, 2018). The application of PGRs to reduce Cd uptake and alleviate Cd toxicity and to boost yield and quality attributes might be a cost-effective, practical, sustainable strategy for MAPs. External supplementation of PGRs is considered an effective practice for reducing the toxic effects of Cd contamination in different crop plants (Rizwan et al., 2016; Zaid and Mohammad, 2018). We recently showed that the external application of PGRs and mineral nutrients had a synergistic effect on alleviating the negative effect of Cd stress on menthol mint plants (Zaid and Mohammad, 2018; Zaid et al., 2020). The incorporation of *M. arvensis* biochar in the soil increased HM tolerance in *M. arvensis* in terms of improving yield and nutrient uptake (Nigam et al., 2019). The concomitant application of depolymerized polysaccharides and PGRs modulated physiological activities, active constituents, and EO production in menthol mint plants (Naeem et al., 2017). Bose et al. (2013) studied the synergistic effect of PGRs and diterpenoid on growth attributes, trichome size, density, and number, EO biosynthesis and the expression of some oil biosynthetic pathway genes. The effect of exogenous treatment of SA on growth, photosynthetic traits and EO production of peppermint plants exposed to soil-applied Cd was studied and the results advocated the ameliorative role of SA (Ahmad et al., 2018).

Seed priming before sowing is an important strategy to ensure optimum hydration for seeds without enabling radical emergence (Hussain et al., 2019b). Priming tends to enhance plant tolerance to abiotic stress by improving plant growth and cellular homeostasis under various stress conditions (Jisha et al., 2013; Savvides et al., 2016). SA is a potent PGR known to impart stress resistance in diverse crop plants (Khan et al., 2015; Ahmad et al., 2019; Santisree et al., 2020). Studies have shown that priming with PGRs is a promising field in plant stress physiology and crop stress management, which can overcome the toxic impacts of abiotic stresses in plants (Lal et al., 2018; Moulick et al., 2018; Panuccio et al., 2018; Sheteiwy et al., 2018). Priming with PGRs is regarded as an eco-friendly, cost-effective, and sustainable practice for reducing the effects of HM stress in plants (Sytar et al., 2018). Hassan and Mansoor (2017) showed that seed priming with PGRs alleviated Cd toxicity in mungbean. Priming with SA could boost growth, photosynthetic capacity and ameliorate the ill effects of HM stress in plants. For example in maize, Zanganeh et al. (2018) studied the coordinated role of seed priming with SA and sodium hydrosulfide under Pb stress. Shahnawaz et al. (2017) studied the impact of Al toxicity on barley cultivars and its alleviation through seed priming with ascorbic acid and SA. In a recent study, we identified the potential of exogenous SA for overcoming nickel stress in Indian mustard plants (Zaid et al., 2019b). However, the role of SA *via* priming under Cd stress in *M. arvensis* in relation to SM modulation and physio-biochemical traits has not been evaluated till date. Therefore, the present study aimed to determine (a) the effects of Cd stress on growth traits, photosynthetic potential, stomatal conductance, and yield and quality traits, (b) the potential of priming suckers with SA to mitigate the adverse impacts of Cd stress, and (c) whether SA priming enhances SM production in menthol mint plants in Cd-contaminated soils.

## Materials and Methods

### Sucker Priming and Stress Treatment

The experiment was performed in a net house at the Botany Department, Aligarh Muslim University Aligarh, India (27°52′N, 78°51′E, 187.45 m altitude), with average day/night temperatures of 26 ± 3/14 ± 2°C, 65 ± 5% relative humidity, 815 ± 20 μmol m^−2^ s^−1^ photosynthetically active radiation (PAR), and 10–12 h critical photoperiod. Uniform suckers of *M. arvensis* Linn. “Kushal” were hand-picked for surface sterilization with 5% NaOCl solution for 6 min, followed by repeated washing with deionized and double distilled water (DDW). The suckers were primed with a solution of SA (10^−6^ M) in the dark at room T for 30 min and then transplanted to earthen pots. Each pot (25 × 25 cm) contained a 5 kg mixture of soil and farmyard manure (4:1). The physicochemical characteristics of the soil were sandy loam texture, pH (1:2) 7.9, EC (1:2) 0.58 mmhos cm^−1^, and 96.8, 9.6, and 158.91 mg kg^−1^ soil available N, P, and K, respectively. A basal dose of N, P, and K (N_100_P_50_K_50_; Khanuja et al., 2006) was applied, being 44.6 mg N, 22.3 mg P_2_O_5_, and 27.3 mg K_2_O kg^−1^ soil as urea, single superphosphate and muriate of potash, respectively. After germination, plants were thinned, and three healthy seedlings were maintained in each pot. Thirty days after transplanting (DAT), two Cd treatments (50 and 100 μm) were applied to pots. A dilute stock aqueous solution of cadmium chloride (CdCl_2_) was prepared by dissolving 1.63 g of CdCl_2_ in 100 ml of DDW and then the required quantity prepared by further diluting the stock solution with DDW. Control plants were provided with DDW only. The experimental pots were positioned in a randomized complete block design. In all, there were six treatments with five replications (*n* = 5): Control (CN; 0 μm Cd + 0 M SA); Salicylic acid (SA 10^−6^ M + Cd 0 μm); Cadmium (50 μm + 0 M SA); Cadmium + Salicylic acid (50 μm + 10^−6^ M SA); Cadmium (100 μm + 0 M SA); Cadmium + Salicylic acid (100 μm + 10^−6^ M SA). The plants were harvested at 100 DAT. Biochemical parameters and antioxidant enzyme activities of the menthol mint plants were determined in the topmost fully expanded young leaves.

### Determination of Growth Parameters

Plant height (PH) was measured with a scale. Plant fresh and dry weights were weighed by using an electronic balance (Verbal 100 Super, Varanasi, Balance Works, Varanasi, India). After recording fresh weight, samples were oven-dried for dehydration at 70°C for 24 h and then weighed. The number of leaves per pot was counted manually. Leaf area was determined using a leaf area meter (AM 350, ADC Bioscientific, Hoddesdon, Herts, United Kingdom).

### Determination of Photosynthetic Pigments

Photosynthetic pigment determination followed the protocol of Lichtenthaler and Buschmann (2001). Fresh leaf tissue (100 mg) was harvested and ground with 100% acetone (v/v) using a deep freezer cooled mortar and pestle. The absorbance of the pigment solution was recorded at 663, 645, and 470 nm to determine the contents of chlorophyll a, chlorophyll b, and carotenoids, respectively, using a spectrophotometer (ELICO SL 171 MINI SPEC) and dimethyl sulfoxide (DMSO) as a blank. Total chlorophyll (Chl) content was estimated by adding the contents of chlorophyll a and b. All pigments were expressed as mg g^−1^ leaf FW.

### Determination of Leaf Gas Exchange Attributes

Leaf gas exchange measurements [net photosynthetic rate (*P*_N_), stomatal conductance (*g*_s_), carbon dioxide assimilation rate (*Ci*), and water use efficiency (WUE)] were measured on the uppermost fully expanded leaves in full and bright sunlight between 10:00 h and 12:00 h using IRGA (Li-COR 6400, Li-COR, Lincoln, NE, United States).

### Determination of Oxidative Stress Biomarkers

The superoxide (O_2_^–•^) content was determined by estimating nitrite production from the reaction of hydroxylamine with superoxide anions by adopting the standard method of Wang and Luo (1990). A 1 g leaf sample was homogenized in phosphate buffer (50 mm, pH 7.8) and polyvinylpyrrolidone solution and centrifuged for 20 min at 4°C. The supernatant was mixed with potassium phosphate buffer and hydroxylamine hydrochloride (50 mm, pH 7.8), then dissolved in a 3-aminobenzenesulphonic acid (17 mm) and 1-naphthylamine (7 mm) and incubated for 20 min at room T. The absorbance was measured at 530 nm and the O_2_^–•^ generation rate calculated from the standard curve of sodium nitrite and expressed as μmol g^−1^ leaf FW.

Thiobarbituric acid reactive substances (TBARS) content was determined by estimating the MDA equivalents following the method of Hodges et al. (1999). Liquid N_2_ frozen leaf tissue (0.5 g) was homogenized in 80% ethanol. The homogenate was centrifuged at 3000 *g* for 10 min. The supernatant (1 ml) was removed and added to an equal volume of 20% trichloroacetic acid (TCA), 0.65% thiobarbituric acid (TBA), and 0.01% butylated hydroxytoluene. The samples were heated at 90°C for 30 min, followed by gradual cooling to room T. Absorbance of the samples was read at 440, 532, and 600 nm, relative to a blank sample with no TBA present, to record MDA concentration.

The method of Sullivan and Ross (1979) was used to determine electrolyte leakage (EL); the detailed protocols described in Zaid and Mohammad (2018). Electrolyte leakage was calculated as follows:

Electrolyte leakage (%) = [(ECb–ECa)/(ECc) × 100].

The standard method of Jana and Choudhari (1981) was used for H_2_O_2_ determination. A fresh leaf sample (500 mg) was homogenized in 3.0 ml of phosphate buffer (50 mm and pH 6.8) and centrifuged at 6000 *g* for 30 min. After that, 3.0 ml of extract was removed and 1 ml of 0.1% titanium chloride in 20% (v/v) H_2_SO_4_ added to it. Centrifugation was carried out at 6000 *g* for 15 min. The absorbance of the reaction mixture was read at 410 nm on a spectrophotometer (ELICO SL 171 MINI SPEC, India).

### Estimation of Antioxidant Activities

Freshly collected leaf material (500 mg) was frozen and ground to a powder using liquid N_2_ in a pre-chilled mortar and pestle. After then, the samples were centrifuged at 12,000 *g* for 30 min at 4°C. The pellet was discarded and the supernatant used to determine different enzyme activities.

Superoxide dismutase activity (SOD, EC1.15.1.1) was measured using the nitroblue tetrazolium (NBT) reduction method (Kono, 1978). Catalase (CAT, 1.11.1.6) activity was determined by monitoring the disappearance of H_2_O_2_ for 2 min at 240 nm (Aebi, 1984). The complete methodology for SOD and CAT determination is described elsewhere (Khanam and Mohammad, 2018). Ascorbate peroxidase (APX, 1.11.1.11) activity was determined using the protocol of Nakano and Asada (1981). Glutathione reductase (GR, 1.6.4.2) activity was measured according to the method of Smith et al. (1988). Enzyme activities were expressed in enzyme units g^−1^ FM. All spectrophotometric analyses were conducted with a spectrophotometer (ELICO SL 171 MINISPEC).

### Determination of Proline, Glycine Betaine, and Leaf Mineral Nutrients

Proline (Pro) and glycine betaine (GB) contents were determined according to the methods of Bates et al. (1973) and Grieve and Grattan (1983), respectively.

For mineral nutrient determinations, oven-dried leaf material (100 mg) was added to a 50 ml Kjeldahl flask containing an acid-peroxide solution and heated on a dry block heater. Aliquots of this solution were used to determine leaf N, P, and K contents. Leaf N content was determined using the protocol of Lindner (1944). The absorbance of the reaction mixture was read at 525 nm. Leaf P content was determined following the methodology of Fiske and Subbarow (1925). The intensity of the blue color formed was recorded at 620 nm. Leaf K content was determined by flame emission spectroscopy using a flame photometer (Model, ELICO, CL22D, India), running at 10 psi pressure (Hald, 1947).

### Scanning Electron Microscopy and Energy Dispersive X-ray Analyzer

The method of Zaid and Mohammad (2018) was used for scanning electron microscopy (SEM) and energy dispersive X-ray analyzer (EDX) analysis. Fresh leaves from axillary positions were collected and fixed with 2.5% glutaraldehyde plus 2% paraformaldehyde in 0.1 M phosphate buffer (pH 7.0) for 4 h. This was followed by a second fixing with 1% osmium tetroxide in P buffer (pH 7.0) for 1 hand washing with P buffer for 20 min. Fixation was followed by alcohol dehydration in a graded series of ethanol (50, 70, 80, 90, 95, and 100%). Fixed and dehydrated leaves were dehydrated in a Carl Zeiss EVO 40 (Germany) scanning electron microscope critical point dryer with liquid CO_2_. The leaf samples were coated with gold–palladium and observed under a Carl Zeiss EVO 40 (Germany) scanning electron microscope at high voltage (15 kV) and magnifications of 4,000 × for stomata and 100 × for peltate glandular trichomes (PGT).

### Estimation of Essential Oil Content and Secondary Metabolites by Gas Chromatography

Essential oil (EO) content was determined following the hydro-distillation technique of Guenther (1972), whereby 50 g of freshly collected leaves were allowed for 3 h distillation. The oil was collected in vials of Clevenger apparatus and weighed. The oil percentage was calculated as follows: EO percentage = oil content/leaf weight × 100. Secondary metabolite (menthol, menthone, and methyl acetate) contents of the EO were determined by gas chromatography (GC) using the known peak area in the chromatogram (Adams, 2007). The estimation of SMs used a gas chromatograph (Nucon 5,700, New Delhi, India) equipped with an AT-1000 stainless steel column, flame ionization detector, and integrator. N gas was used as the carrier. N, H, and O flow rates were adjusted at 0.5, 0.5, and 5mLs^−1^, respectively. The sample size was 2 μl. The T recorded was 160°C oven, 250°C detector, and 250°C injector.

### Statistical Analysis

Data were analyzed following one-way analysis of variance (ANOVA) using SPSS software version 17. The values presented are means ± SE (*n* = 5). Different letters indicate significant differences between treatments at *p* ≤ 0.05. Principal component analysis was performed using Minitab Mtb EXE (2) software (Minitab Inc., State College PA, United States).

## Results

### Effect of Salicylic Acid on Plant Growth Parameters Under Cadmium Stress

The Cd treatments significantly decreased (*p ≤ 0.05*) plant fresh and dry weights, by 18.16 and 40.84% at 50 μm and 8.52 and 26.13% at 100 μm, respectively, relative to the control (Table 1). The SA + Cd treatments increased plant fresh weights by 7.57 and 26.66% and dry weights by 20.94 and 12.08% at 50 and 100 μM, respectively, relative to the respective Cd-only treatments. PH declined significantly (*p ≤ 0.05*) by 10.25 and 17.70%, relative to the control plants. Priming with SA increased PH in Cd-stressed plants by 6.56 and 7.37% at 50 and 100 μm, respectively, relative to the respective Cd-only treatments (Table 1).

**Table 1:** Plant fresh and dry weights (g), plant height (cm), and leaf number and area per plant, of *Mentha arvensis* cultivar “Kushal” grown under 50 and 100 μm Cd levels and primed with or without salicylic acid.

Treatments	Plant fresh weight (g)	Plant dry weight (g)	Plant height (cm)	Leaf/plant	Area/leaf (cm^2^)
CN	65.00 ± 2.90b	26.29 ± 1.29ab	79.93 ± 1.63b	15.20 ± 0.91b	7.75 ± 1.17b
SA (10^−6^ M)	75.90 ± 1.22a	31.47 ± 1.43a	88.04 ± 1.35a	20.00 ± 1.88a	9.31 ± 1.18a
Cd 50 (μm)	53.19 ± 0.69c	24.05 ± 1.03bc	71.73 ± 1.03c	13.20 ± 1.47b	6.01 ± 0.56 cd
Cd 50 (μm) + SA (10^−6^ M)	57.55 ± 2.22c	30.42 ± 0.89a	76.77 ± 1.43b	15.40 ± 1.51b	7.72 ± 1.01b
Cd 100 (μm)	38.45 ± 2.57d	19.42 ± 1.29c	65.78 ± 1.48d	7.80 ± 1.21c	4.70 ± 0.77d
Cd 100 (μm) + SA (10^−6^ M)	52.43 ± 1.88c	22.09 ± 1.31bc	71.02 ± 0.92c	12.60 ± 1.06b	6.44 ± 1.07bc

*Data are presented as treatments mean ± SE (*n* = 5). Data followed by the same letters are not significantly different by Duncan multiple range test at *p* ≤ 0.05*.

Leaf number declined by 13.15 and 48.68% at 50 and 100 μm, respectively, relative to the control plants. Priming with SA increased leaf number by 14.28 and 38.09% at 50 and 100 μm, respectively, relative to the respective Cd-only treatments. Leaf area also declined significantly (*p ≤ 0.05*) by 22.45 and 39.35% at 50 and 100 μm, respectively, relative to the control plants. Priming with SA increased leaf area by 22.15 and 27.01% at 50 and 100 μm, respectively, relative to the respective Cd-only treatments (Table 1).

### Salicylic Acid Restored Photosynthetic Pigments and Gas Exchange Attributes

The Cd treatments had a negative effect on photosynthetic pigments (Chl a, b, total Chl, and carotenoids). Chl a and b contents declined by 7.94 and 5.71% at 50 μm and 15.23 and 20% at 100 μm, respectively, relative to the control plants. Total Chl and carotenoid content also declined by 7.52 and 16.12% at 50 μm and 3.22 and 16.12% at 100 μm, respectively, relative to the control plants (Table 2). Priming with SA restored the photosynthetic pigment inhibition, increasing Chl a by 2.79 and 3.03%, Chl b by 13.15 and 12.50%, total Chl by 4.79 and 4.87%, and Caro content by 6.25 and 7.14% at 50 and 100 μm, respectively, relative to the respective Cd-only treatments (Table 2).

**Table 2:** Contents of chlorophyll a, b, total chlorophyll, and carotenoids (mg g^−1^FW) of *Mentha arvensis* cultivar “Kushal” grown under 50 and 100 μm Cd levels and primed with or without salicylic acid.Data are presented as treatments mean ± SE (*n* = 5).

Treatments	Chlorophyll a (mg g^−1^FW)	Chlorophyll b (mg g^−1^FW)	Total Chlorophyll (mg g^−1^FW)	Carotenoids (mg g^−1^FW)
CN	1.51 ± 0.01b	0.35 ± 0.07b	1.86 ± 0.04b	0.31 ± 0.01b
SA (10^−6^ M)	1.62 ± 0.02a	0.40 ± 0.07a	2.02 ± 0.01a	0.37 ± 0.01a
Cd 50 (μm)	1.39 ± 0.01c	0.33 ± 0.05bc	1.72 ± 0.01c	0.30 ± 0.07bc
Cd 50 (μm) + SA (10^−6^ M)	1.43 ± 0.02c	0.38 ± 0.09a	1.81 ± 0.03b	0.32 ± 0.07b
Cd 100 (μm)	1.28 ± 0.01d	0.28 ± 0.01d	1.56 ± 0.01e	0.26 ± 0.00c
Cd 100 (μm) + SA (10^−6^ M)	1.32 ± 0.02d	0.32 ± 0.07c	1.64 ± 0.01d	0.28 ± 0.02bc

*Data followed by the same letters are not significantly different by Duncan multiple range test at *p* ≤ 0.05*.

**Figure 1:**
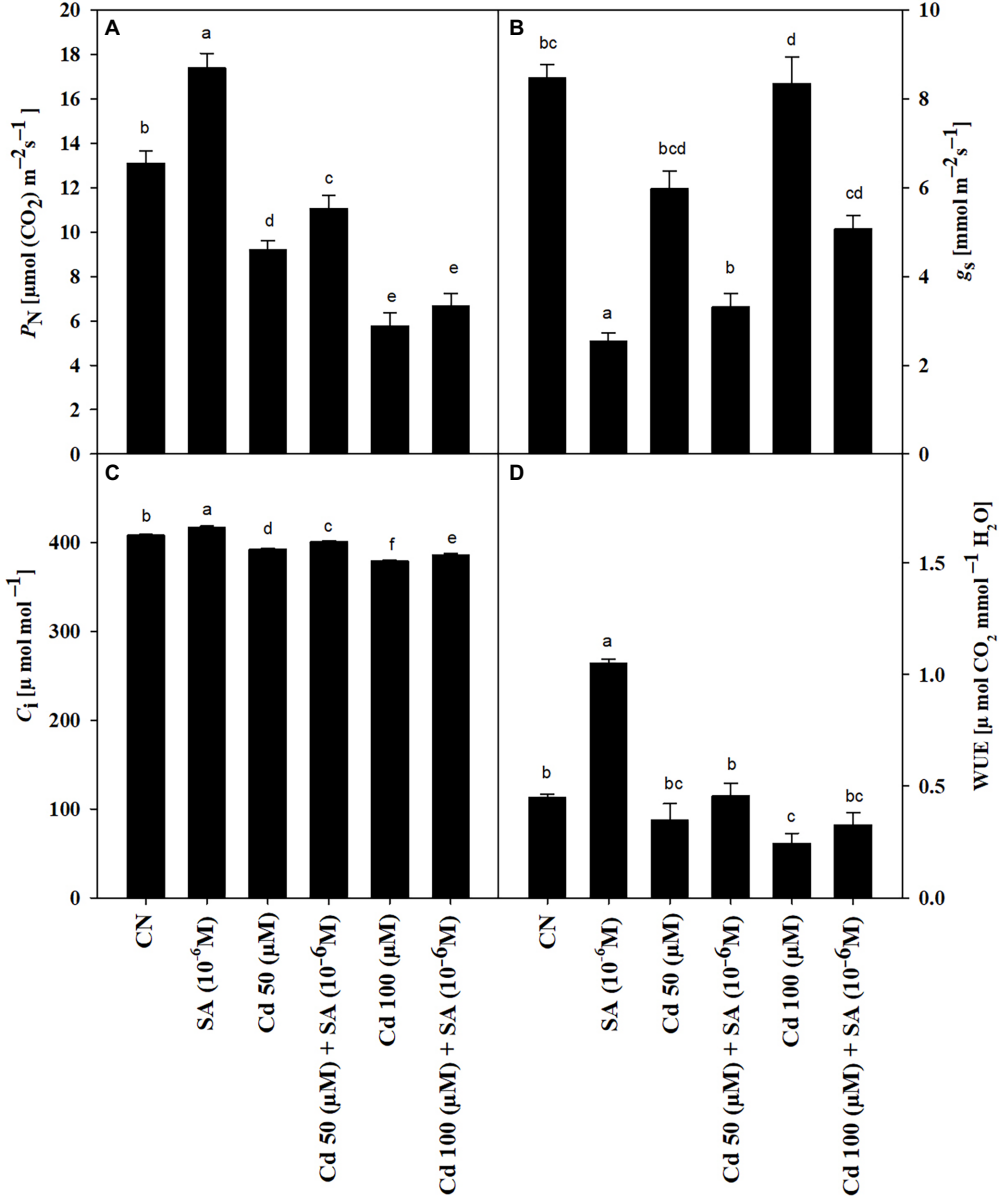
**(A)** Net photosynthetic rate, *P*_N_ (μmol CO_2_ m^−2^ s^−1^), **(B)** stomatal conductance, *g*_s_ (mmol m^−2^ s^−1^), **(C)** intercellular CO_2_ concentration, *C*_i_ (μmol mol^−1^), and **(D)** water use efficiency, WUE (μmol CO_2_ mmol^−1^ H_2_O) of *Mentha arvensis* cultivar “Kushal” grown under 50 and 100 μm Cd levels and primed with or without salicylic acid. Data are presented as treatments mean ± SE (*n* = 5). Data followed by the same letters are not significantly different by Duncan multiple range test at *p* ≤ 0.05.

The Cd treatments reduced P*_N_* by 29.47 and 55.88% at 50 and 100 μm, respectively, relative to the control plants. Priming with SA significantly improved P*_N_* by 16.83 and 13.23% at 50 and 100 μm, respectively, relative to the respective Cd-only treatments (Figure 1A). Priming with SA reversed the decline in g*s* and Ci caused by Cd stress to values 3.35 and 1.89% at 50 μm and 3.85 and 1.83% at 100 μm higher than the respective Cd-only treatments (Figures 1B,C). The 50 and 100 μm Cd treatments decreased WUE by 24.44 and 46.66%, respectively, relative to the control plants. Priming with SA increased WUE by 24.44 and 25% at 50 and 100 μm, respectively, relative to the respective Cd-only treatments (Figure 1D).

### Salicylic Acid Reduced Thiobarbituric Acid Reactive Substance, Electrolyte Leakage, Hydrogen Peroxide Content, and Superoxide Ion Production Under Cadmium Stress

**Figure 2:**
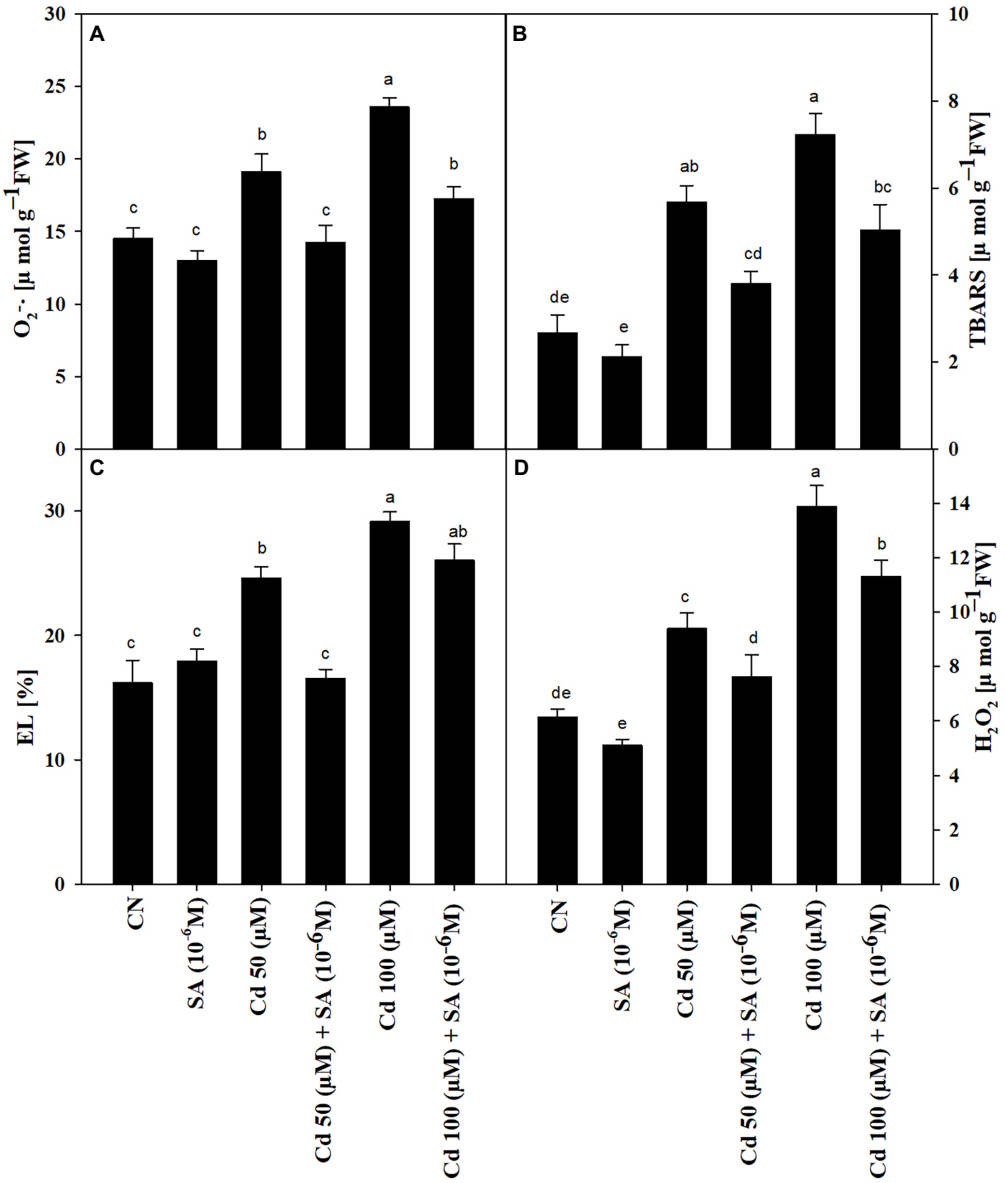
**(A)** Superoxide, O_2_^–·^ (μmol g^−1^ FW), **(B)** thiobarbituric acid reactive substances, TBARS (μmol g^−1^ FW), **(C)** electrolyte leakage, EL (%), and **(D)** hydrogen peroxide, H_2_O_2_ (μmol g^−1^ FW) of *Mentha arvensis* cultivar “Kushal” grown under 50 and 100 μm Cd levels and primed with or without salicylic acid. Data are presented as treatments mean ± SE (*n* = 5). Data followed by the same letters are not significantly different by Duncan multiple range test at *p* ≤ 0.05.

The Cd treatments increased TBARS content, EL, H_2_O_2_ content_,_ and O_2_^–•^ production by 53.08, 34.21, 34.71 and 24.07% at 50 μM, respectively, relative to the control (Figures 2A–D), which was intensified further at 100 μM. Priming with SA significantly reduced TBARS content by 32.98%, EL by 32.83%, H_2_O_2_ content by 19.06%, and O_2_^–•^ production by 25.58% in the 50 μm Cd treatment, relative to respective Cd-only treatment. At 100 μm Cd, SA priming decreased TBARS content by 30.33%, EL by 10.47%, in H_2_O_2_ content by 18.60% and O_2_^–•^ production by 26.77%, relative to respective Cd-only treatment (Figures 2A–D).

### Salicylic Acid Enhanced Antioxidant Enzyme Activities

**Figure 3:**
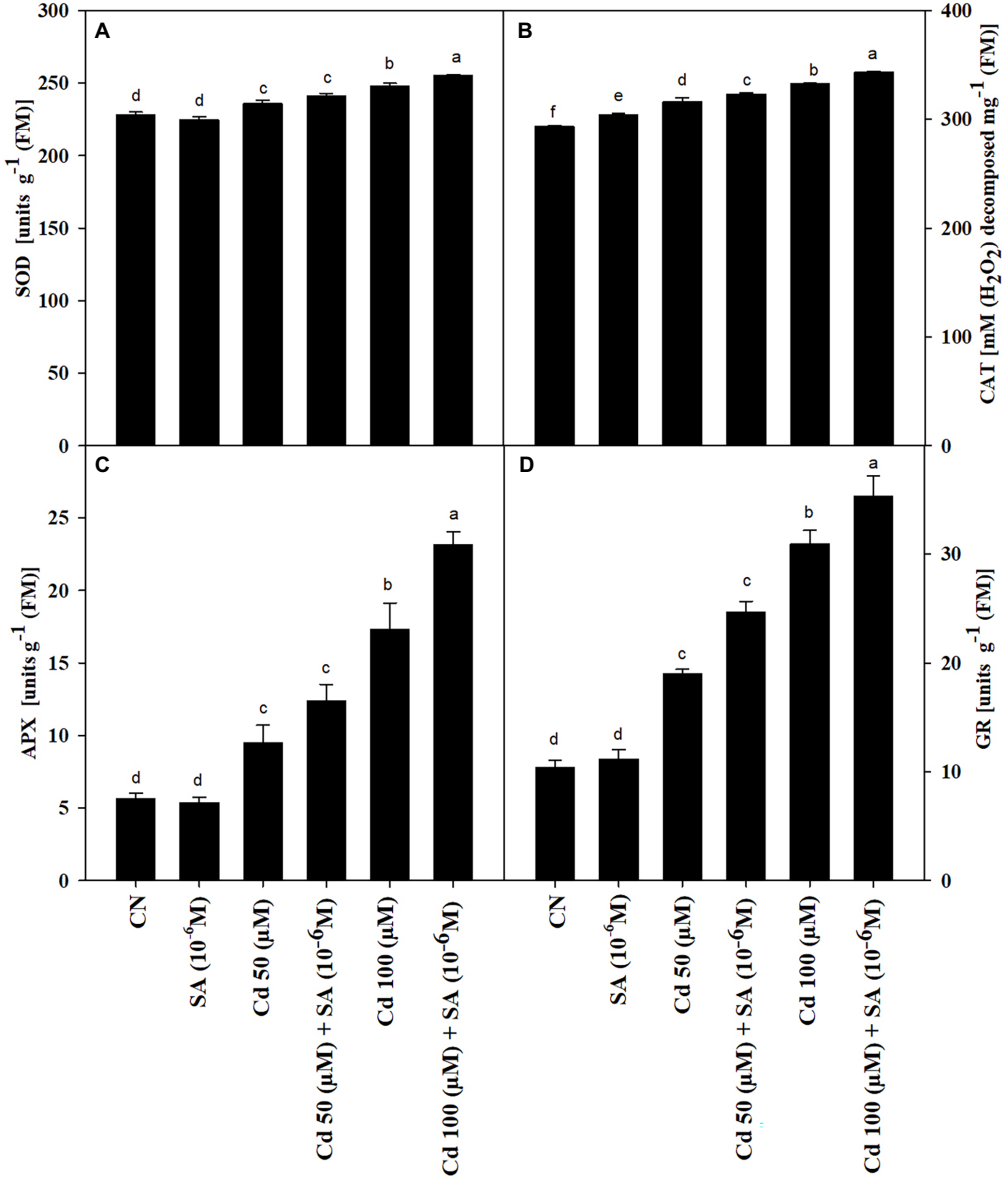
Activities of **(A)** superoxide dismutase, SOD (units g^−1^ FM), **(B)** catalase, CAT (units g^−1^ FM), **(C)** ascorbate peroxidase, APX (units g^−1^ FM), and **(D)** glutathione reductase, GR (units g^−1^ FM) of *Mentha arvensis* cultivar “Kushal” grown under 50 and 100 μm Cd levels and primed with/without salicylic acid. Data are presented as treatments mean ± SE (*n* = 5). Data followed by the same letters are not significantly different by Duncan multiple range test at (*p* ≤ 0.05).

The Cd treatments increased the activities of SOD, CAT, APX, and GR by 3.27 and 7.90%, 7.27 and 11.79%, 40.73 and 67.47%, 30.32 and 56.57% at 50 and 100 μm, respectively, relative to the control plants. Priming with SA had an additive impact on SOD, CAT, APX, and GR activities, which increased by 2.18 and 2.88%, 2.13 and 3.09%, 23.20 and 25.16%, and 9.23 and 12.88% at 50 and 100 μm, respectively, relative to the respective Cd-only treatments (Figures 3A–D).

### Effect of Salicylic Acid and Cadmium on Osmoprotectants and Mineral Nutrient Contents

**Figure 4:**
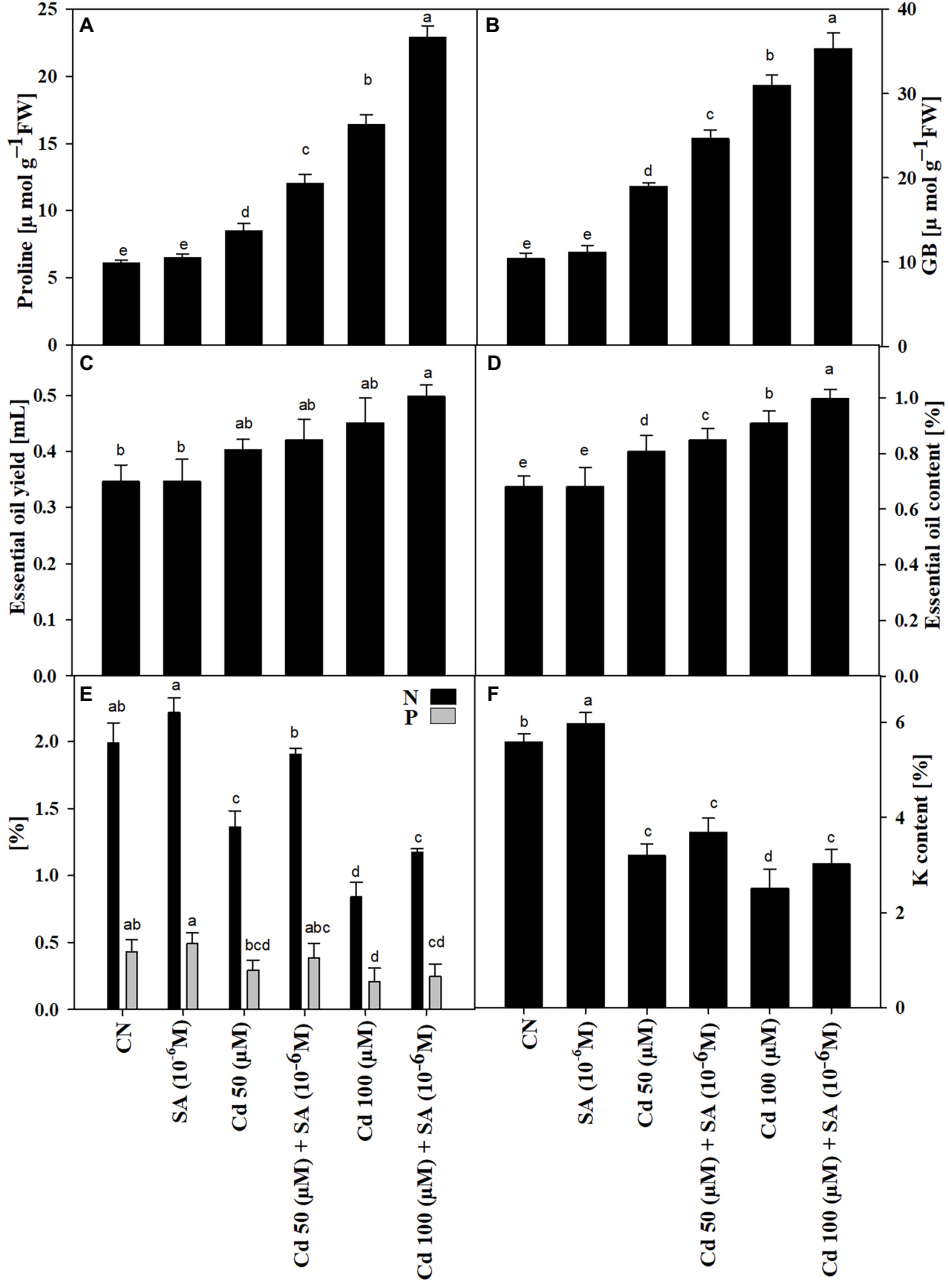
Contents of **(A)** proline (μmolg^−1^ FW), **(B)** glycine betaine, GB (μmolg^−1^ FW), **(C)** essential oil yield, EO (mL), **(D)** essential oil content, EO (%), **(E)** nitrogen and phosphorous (%), and **(F)** potassium content (%) of *Mentha arvensis* cultivar “Kushal” grown under 50 and 100 μm Cd levels and primed with or without salicylic acid. Data are presented as treatments mean ± SE (*n* = 5). Data followed by the same letters are not significantly different by Duncan multiple range test at *p* ≤ 0.05.

The Cd treatments triggered the accumulation of pro and GB in the presence or absence of SA. The pro and GB contents increased significantly (*p ≤ 0.05*) by 1.39-fold and 1.83-fold, and 2.69-fold and 2.98-fold, at 50 μm and 100, respectively, relative to the control plants. Priming with SA increased pro and GB contents by 1.41-fold and 1.29-fold at 50 μm and 1.39-fold and 1.41-fold at 100 μm, relative to the respective Cd-only treatments (Figures 4A,B).

The Cd treatments decreased the mineral nutrient (N, P, and, K) contents, relative to the control plants. Priming with SA increased N content by 28.42%, P by 23.68%, and K by 13.55% at 50 μm and N by 28.20%, P by 16.94% and K (not significant) by 16.66% at 100 μm, relative to the respective Cd-only treatments (Figures 4E,F).

### Salicylic Acid Reduced Cadmium Content as Evidenced by EDX

**Figure 5:**
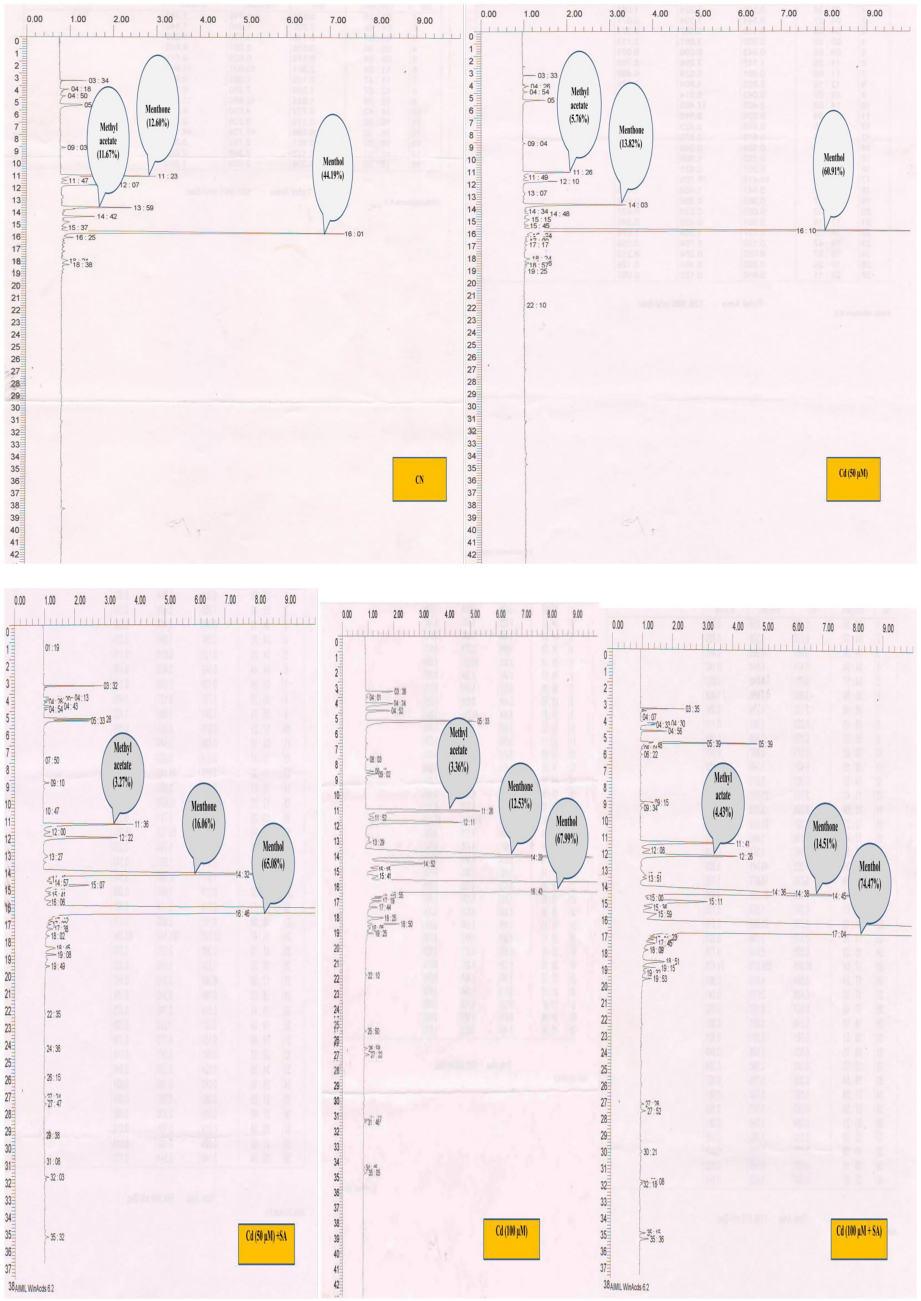
Cadmium-mediated effect of salicylic acid (SA) sucker priming on menthol, menthone and methyl acetate content (GC graph) of *Mentha arvensis* cultivar “Kushal” in different treatments. GC chromatograms showing the peaks of different components of the essential oil. Abbreviations: CN, control; Cd, cadmium.

The plants in the 50 and 100 μm Cd treatments accumulated 97.95 and 98.03% more Cd, respectively, than the control plants. Priming with SA reduced Cd content by 79.59 and 29.41% at 50 and 100 μm, respectively, relative to the respective Cd-only treatments (Figure 5D).

### Salicylic Acid and Cadmium Enhanced Essential Oil Content and Secondary Metabolite Production

The Cd treatments increased EO content and EO yield. Priming with SA had an additive effect, increasing EO content by 4.76% and EO yield by 8.16% at 50 and 100 μm, respectively, relative to the respective Cd-only treatments (Figures 4C,D).

**Figure 6:**
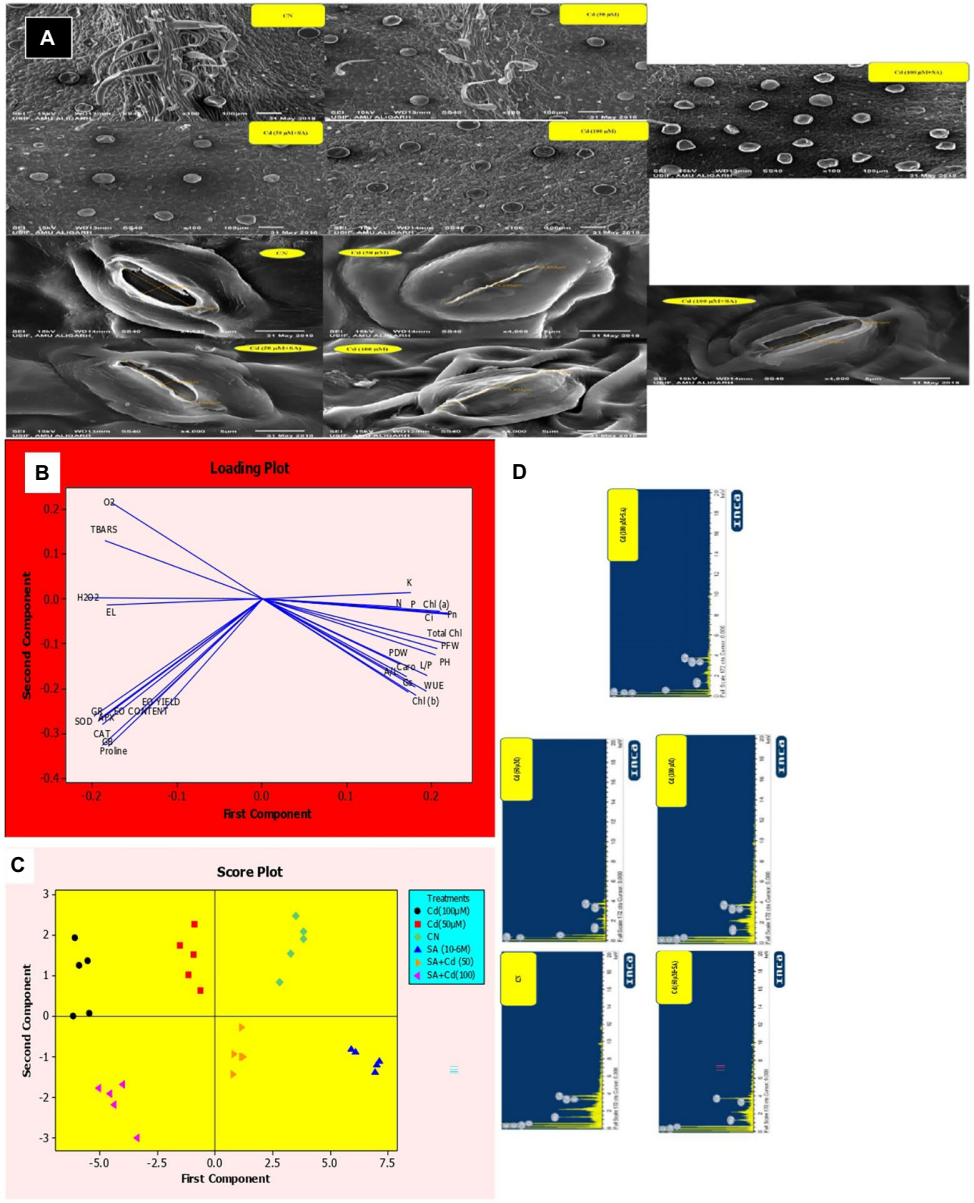
**A**: Cadmium-mediated effect of salicylic acid (SA) sucker priming on peltate glandular trichome (PGT) density of *Mentha arvensis* cultivar “Kushal” at different treatments. Abbreviations: CN, control; Cd, cadmium. Stomatal behavior of *M. arvensis* leaves in different treatments. The opening and closing of stomatal behavior were observed under a scanning electron microscope at 4000 × magnification in *M. arvensis* leaves treated with 50 and 100 μm CdCl_2_ at 30 days after transplantation (DAT). A single representative of each treatment is shown. Principal component analysis (PCA) of various parameters of menthol mint plants under cadmium (Cd) and salicylic priming (SA), **(B)** Loading plot of different parameters showing positive and negative correlations, **(C)** Score plot differentiating the treatments. PFW, plant fresh weight; PDW, plant dry weight; PH, plant height; L/P, number of leaves per plant; A/L, area per leaf; Chl (a), chlorophyll a; Chl (b), chlorophyll b; Total Chl, total chlorophyll; Caro, carotenoids; *P_N_*, net photosynthetic rate; *gs*, stomatal conductance; *Ci*, intercellular CO_2_ concentration; WUE, water use efficiency; O_2_, superoxide ion; TBARS, thiobarbituric acid reactive substances; EL, electrolyte leakage; H_2_O_2_, hydrogen peroxide; SOD, superoxide dismutase activity; CAT, catalase; APX, ascorbate peroxidase; GR, glutathione reductase; GB, glycine betaine; EO, essential oil; N, nitrogen; P, phosphorous; K, potassium, and **(D)** EDX spectrum showing the content of Cd of *M. arvensis* cultivar “Kushal” at different treatments. Abbreviations: CN, control; Cd, cadmium.

Priming with SA triggered the production of SM in menthol mint plants in both Cd treatments; 50 μm Cd increased menthol content by 6.40% and menthone by 13.94% but decreased methyl acetate, and 100 μm Cd increased menthol content by 8.70%, menthone by 13.64%, and methyl acetate by 24.15%, relative to the respective Cd-only treatments (Figure 6).

### Effect of Salicylic Acid and Cadmium on Stomatal Response and Peltate Glandular Trichome Density Under Electron Microscopy

SA enriched the number of PGT in menthol mint plants, progressively from the control to the 100 μm Cd + SA treatment (Figure 5A).

Analysis of SEM showed that the Cd treatments induced stomatal closure. The control plants had a normal and open stomatal aperture with 3.08 μm pore size and characteristic guard cells, while the stomata in the 50 and 100 μm Cd treatments had partially closed with 0.25 μm and 1.52 μm pore size, respectively. Priming with SA decreased the Cd effects to open stomata with 1.68 μm and 2.73 μm pore size in the 50 and 100 μm Cd treatments, respectively (Figure 5A).

## Discussion

Cadmium contamination of agricultural land has become a principal constraint to crop production (Zaid and Mohammad, 2018; Hussain et al., 2019a; Rizwan et al., 2019a) as it substantially increases oxidative stress, thereby reducing nutritional homeostasis, growth and metabolism, photosynthetic yield, and quality and quantity of crops (Rizwan et al., 2016, 2019b; Zaid and Mohammad, 2018; Greco et al., 2019). This study was conducted on menthol mint plants to unravel various mechanisms underlying priming with SA that improve Cd-inhibited growth, photosynthesis and yield, and quality attributes.

Significant alterations in growth and photosynthesis parameters were observed in menthol mint plants under Cd stress. Similarly, reduced growth and photosynthesis under Cd stress have been observed in peppermint (Ahmad et al., 2018), wheat (Hussain et al., 2019b), tomato (Alyemeni et al., 2018), and *Lolium perenne* (Zhang et al., 2018). Plant height, fresh and dry weights, and leaf number and area declined in the Cd treatments. The reduced growth and biomass in Cd-treated plants might be because Cd obstructs the progression of the cell cycle and irreversibly alters the proton pumps thereby disrupting membrane permeability (Gomes-Junior et al., 2006; Liu et al., 2015). Thus, the Cd-induced disruptions in membrane permeability may have hindered ion absorption, as reflected in the reduced N, P, and K contents, and thereby hampered plant growth. Priming with SA overcame the Cd-induced growth inhibition. Under Cd stress, SA-induced expression of genes and maintains optimum endogenous SA accumulation that potentiated the Cd-induced growth inhibition (Wang et al., 2019). SA can also reduce the endogenous Cd content and various ROS (Zhang et al., 2015). Thus, the optimum redox balance maintained by SA in the present study under Cd stress increased growth and biomass yield in menthol mint plants.

The values of photosynthetic pigments (Chl a, b, total Chl, and Caro contents) and leaf gas exchange attributes (P_N_, gs, Ci, and WUE) progressively declined with increasing levels of soil-applied Cd, relative to the control plants (Table 2; Figure 1). The Cd-induced inhibition of chlorophyll biosynthesis is due to the retention of Cd^2+^ ions in the thylakoids and stroma of the chloroplasts (Lysenko et al., 2018), and the substitution of Mg^2+^ bound into the chlorophyll molecule (Rydzyński et al., 2018). The reduction in leaf gas exchange attributes at both levels of Cd stress was due to stomatal inhibition (Figure 5A) that restricted gas exchange between the environment and menthol mint plants, and these results are in line with those of Zaid and Mohammad (2018). Priming with SA increased the leaf gas exchange values and nullified the reduction in photosynthesis. Under Cd stress, continuous gas exchange (Figure 5A) due to SA priming induced stomatal recovery to maintain proper gas flow.

The increased Cd content (Figure 5D) under Cd stress declined substantially with SA priming, which was reflected in the increased activity of antioxidant enzymes and biosynthesis of osmolytes (proline and GB; Figures 4A,B). The application of SA decreased the accumulation of Cd in two melon cultivars (Zhang et al., 2015). Karimi and Ghasempour (2019) reported that SA restrained Ni content in *Alyssum inflatum*. Cd levels in the present study causes a state of oxidative stress by orchestrating the production of ROS (H_2_O_2_ and O_2_^–•^ Figures 2A,D), ion leakage (EL, Figure 2C) and lipid oxidation (TBARS, Figure 2B). However, there is an increment in the activities of antioxidants (SOD, CAT, APX, and GR) (Figures 3A–D), which scavenge Cd-induced excess ROS and maintain an optimum redox homeostatic state (Zaid and Mohammad, 2018). Under Cd stress, SA triggered the expression of selected genes related to ROS and SA to regain the redox state (Li et al., 2019), which in turn enhanced the tolerance of menthol mint plants to Cd stress. Exogenous supplements of SA under Cd stress can increase endogenous SA (Wang et al., 2019); the enhanced biosynthesis of SA in the present study might have scavenged excess ROS and thus lowered TBARS and EL levels in the Cd treatments (Figures 2B,C). Similarly, pre-soaking mungbean seeds in SA solution alleviated Cd-induced toxicity (Roychoudhury et al., 2016). Interestingly, low Cd content in SA-treated plants (Figure 5D) further reduced Cd-induced stress, indicating that the induced up-regulation of redox-related enzymes by SA priming under Cd stress plays a crucial role in scavenging intracellular ROS, thus, highlighting the importance PGRs priming in maintaining redox equilibrium under elevated levels of Cd in menthol mint plants.

Osmolytes, such as proline and GB, are compatible cytosolutes with modulatory roles in plants exposed to metal stress, including detoxifying ROS and chelating HM ions (Ashraf and Foolad, 2007; Bharwana et al., 2014; Zouari et al., 2016). Priming with SA increased the biosynthesis of osmolytes under Cd stress, which was maintained at 100 μM Cd (Figures 4A,B). Increased accumulation of osmolytes with SA priming resulted in the detoxification of ROS and chelation of Cd^2+^ ions as evidenced by EDX (Figure 5D). Similar SA-mediated increases in osmolytes under metal stress have been reported in crop plants including *Alyssum inflatum* (Karimi and Ghasempour, 2019), *Melissa officinalis* (Soltani Maivan et al., 2017), *Triticum aestivum* (Alamri et al., 2018), and *Zea mays* (Zanganeh et al., 2018).

Mineral nutrient contents (N, P, and, K) decreased as Cd levels increased from 50 to 100 μm (Figures 4E,F). Cd restricts the uptake of these important mineral elements (Zaid and Mohammad, 2018). In the present study, Cd contamination increased the endogenous Cd content that restricts mineral uptake because excess Cd^2+^ ions impose antagonistic effects on ion uptake (Nazar et al., 2012). Priming with SA restored mineral contents by either directly increasing their content or indirectly decreasing Cd^2+^ ions. Our study corroborates the findings of Ahmad et al. (2011). In bean plants exposed to short-term Cd stress, nutrient concentrations also increased in the SA treatment (Saidi et al., 2013).

Plants produce myriad of SMs, which protect against various environmental pressures (Berini et al., 2018). PGRs, especially SA, mediate a tolerance trade-off by eliciting SM production in plants (Singh and Dwivedi, 2018). HMs alter the production of SMs in plants; we observed significant modulation in menthol, menthone, and methyl acetate under Cd stress (Figure 6). Similarly, Paeizi et al. (2018) observed marked changes in medicinal alkaloid production in response to metal stress under PGR in *Catharanthus roseus*. We also observed an overall increase in EO and SM production with SA priming, which agrees with the findings of Darvizheh et al. (2018) in *Echinacea purpurea*. The increase in SMs by SA was due to an increase in PGT density. As observed in Figure 5A, SA increased the density of PGT under Cd, with the maximum density observed at 100 μM Cd; hence, increased PGT density is directly responsible for increasing EO and SM biosynthesis. SA also triggered SM biosynthesis in a *Taxus baccata* callus culture under glucose-mediated alterations (Sarmadi et al., 2018). In a recent study, Mohammadi et al. (2019) reported SA-induced modulations in EO constituents in different ecotypes of *Thymus* species under water stress. Exogenous PGR-mediated increases in SM production under Cd stress are also due to the up-regulation of transcriptional expressions of genes responsible for their biosynthesis (Chen et al., 2017). Moreover, the sustained increments in growth, photosynthesis, and mineral content of SA-treated plants under Cd stress are likely to culminate in maximized EO and SM contents in menthol mint plants.

Principal component analysis (PCA) identified the relationships between the studied traits, their variation, and whether SA priming can improve Cd-stress resistance and differentiate between the various treatments according to their toxicity and amelioration. Both of the Cd treatments affected plant growth, photosynthetic traits and mineral nutrient contents and they were positively correlated; however, oxidative stress biomarkers, antioxidant enzymes, and EO yield and content, which increased with Cd levels, were also positively correlated and clustered opposite the growth, photosynthetic and nutrient content parameters (Figure 5B). Our hypothesis was further confirmed that Cd-mediated oxidative stress induced repressions in menthol mint plants by affecting growth, photosynthetic traits, and mineral nutrient contents. The most toxic treatment was 100 μm Cd, followed by 50 μm Cd, and both were clustered next to each other. The score plot in PCA distinguishes various treatments according to their toxicities (Figure 6C). Starting from left side, SA under CN was the best treatment. It was followed by CN, SA + Cd (50), Cd (50), SA + Cd (100), and Cd (100). Priming plants with SA ameliorated the Cd-induced overall repressions in menthol mint plants.

## Conclusion

In summary priming of menthol mint with SA plants can act as a low-cost, sustainable option in not only overcoming the Cd-induced adverse effects but also increasing its yield and quality attributes. A representation of the present work that how SA priming can overcome Cd stress and increase yield and quality attributes in menthol mint is presented in graphical abstract.

## Data Availability Statement

The original contributions presented in the study are included in the article/supplementary material; further inquiries can be directed to the corresponding authors.

## Author Contributions

AZ and FM designed the experiment. AZ carried out the experimental analysis, wrote the draft, and revised the manuscript. FM corrected the draft and overall supervised the work. KS critically analyzed the manuscript and edited it for language. All authors contributed to the article and approved the submitted version.

## Conflict of Interest

The authors declare that the research was conducted in the absence of any commercial or financial relationships that could be construed as a potential conflict of interest.

## Publisher’s Note

All claims expressed in this article are solely those of the authors and do not necessarily represent those of their affiliated organizations, or those of the publisher, the editors and the reviewers. Any product that may be evaluated in this article, or claim that may be made by its manufacturer, is not guaranteed or endorsed by the publisher.
